# Applied Interventions in the Prevention and Treatment of Obesity Through the Research of Professor Jane Wardle

**DOI:** 10.1007/s13679-017-0249-8

**Published:** 2017-03-06

**Authors:** Helen Croker, Rebecca J. Beeken

**Affiliations:** Department of Behavioural Science and Health, University College London, 1-19 Torrington Place, London, WC1E 6BT UK

**Keywords:** Obesity treatment, Behavioural therapy (BT), Cognitive behavioural therapy (CBT), Family-based behavioural treatment (FBBT), Genetic feedback, Habit formation theory

## Abstract

**Purpose of Review:**

Obesity presents a challenge for practitioners, policy makers, researchers and for those with obesity themselves. This review focuses on psychological approaches to its management and prevention in children and adults.

**Recent Findings:**

Through exploring the work of the late Professor Jane Wardle, we look at the earliest behavioural treatment approaches and how psychological theory has been used to develop more contemporary approaches, for example incorporating genetic feedback and habit formation theory into interventions. We also explore how Jane has challenged thinking about the causal pathways of obesity in relation to eating behaviour. Beyond academic work, Jane was an advocate of developing interventions which had real-world applications.

**Summary:**

Therefore, we discuss how she not only developed new interventions but also made these widely available and the charity that she established.

## Introduction

The research conducted by Professor Jane Wardle on psychological approaches for managing obesity stemmed from her background as a clinical psychologist with interests in, among other topics, eating disorders. In particular, Jane was interested in binge eating and dietary restraint [1, 2]. For example, she found that binge eating is not necessarily an ‘abnormal’ or pathological behaviour—it occurred in even seemingly ‘normal’ individuals and appeared to occur in response to dietary restriction [2]. This early work led on to her interest in eating behaviours more broadly, and a particular interest in those behaviours that were responsible for weight gain and the increasing prevalence of obesity. Jane applied psychological theories to her work, starting with a recognition that a crucial component of obesity treatment is behavioural modification.

## Behavioural Management of Obesity

Nowadays, it is so well accepted that behavioural therapy (BT) is the standard approach for treating obesity, that it is hard to imagine a time when this was not the case. Of course there are more novel psychological approaches, but BT has for many years remained at the forefront of treatment. For example, NICE recommends that ‘multicomponent interventions are the treatment of choice’ for obesity and these should include a behavioural component [3]. A cursory glance at the list of recommended strategies reveals that they are primarily drawn from standard BT and cognitive behavioural therapy (CBT) programmes, although this is not explicitly stated. More recently, there have been advances in treatments that achieve greater weight loss—bariatric surgery and also pharmacological treatments (the latter at least only being prescribed alongside behavioural management)—but BT remains the treatment of choice for the majority of individuals with excess weight. The first effective treatment approach for obesity based on BT was published by Stuart and colleagues in 1967 [4]. Over the subsequent decade, the number of trials of behavioural treatments increased and these consistently demonstrated that BT was more effective than other treatments [5]. However, there were few data regarding long-term (1-year follow-up) outcomes [6]. Programmes evolved to include more information about the behavioural changes themselves rather than just emphasising behaviour change strategies (i.e. the ‘what’ as well as the ‘how’) [7]. Furthermore, components from cognitive therapy (identifying and modifying unhelpful, or negative, thoughts) were incorporated to create cognitive behavioural therapy (CBT) programmes [8, 9]. A recent systematic review of randomised controlled trials identified 37 studies comparing behavioural weight management treatments with non-behavioural approaches [10]. This review indicated that the majority of behavioural programmes were more effective than the comparison groups (with a pooled mean difference of −2.8 kg in favour of treatment at 12 months).

Despite this enthusiasm for addressing obesity with behavioural treatments, there were concerns that ‘dieting’ could be harmful. These concerns led to a growing body of literature which supported the notion that dietary restriction could cause disordered patterns of eating and compensatory overeating, which could in turn lead to weight regain. Jane carried out several experimental studies and challenged some of these prevailing views. For example, in one study, 20 participants completed measures of restrained eating and took part in four experimental conditions where they were given a low- or high-energy drink. In each case, they were told the drink was either of low or high calorie (i.e. were given correct or incorrect nutritional information) [11]. They then ranked their level of hunger and satiety over a 2-h period and participated in a test meal where they were given ad libitum access to a standardised sandwich lunch. There were no differences between experimental groups for satiety ratings, but those with higher levels of restrained eating rated their level of hunger higher after consuming what they believed to be the lower-calorie drink compared to when they believed they had consumed the high-calorie drink, whereas there was no difference for unrestrained eaters. There were also no differences between groups for the amount of food consumed in the test meal, although overall those with higher levels of restrained eating consumed more food. This indicated that although the restrained eaters were more sensitive than non-restrained eaters to the manipulation of external cues (i.e. rated their hunger higher when they believed they had consumed less calories), this manipulation did not influence their food intake so they had not necessarily lost the ability to regulate appetite. In another study, the results from previous work by others, which had found that restrained eaters consumed more under laboratory taste tests, were extended to show that such tests did not evoke longer-term compensation under ‘normal’ conditions [12].

Jane sought to reconcile these two opposing views—that obesity brought health risks and should be addressed and the ‘non-dieting’ movement with its vehement belief that overweight individuals should not be encouraged to diet at all. Jane’s work on treatment programmes took the concerns about dieting into account but, rather than promoting an entirely ‘non-dieting’ approach, she also recognised the very serious consequences for health caused by obesity. If these two very opposing views could be combined, then overweight individuals could be helped to control their weight without the potential adverse effects of over-restriction. Such an approach could have broad appeal, especially for those who struggle to maintain weight losses but can see benefits from reducing their weight. Jane therefore built on the early development work involving behavioural interventions described above but incorporated elements of non-dieting. In 2000, Jane and colleagues published the results of a randomised controlled trial to evaluate a CBT programme for obesity which incorporated a non-dieting approach while maintaining an emphasis on lifestyle change so as not to ignore the substantial physical health costs of obesity [13•]. In this study, a standard CBT programme was compared to modified-CBT comprising behavioural and cognitive strategies alongside approaches to encourage acceptance of body size and avoidance of restrictive eating. Here modest weight loss was expected but was not the explicit goal of treatment. Participants attended weekly group sessions over 10 weeks and significant, albeit modest, weight loss was achieved by participants in both programmes at the 52-week follow-up. Additionally, there were improvements in a range of other physical (e.g. total, HDL and LDL cholesterol, blood pressure and waist circumference) and psychological (mood, self-esteem, stress, binge eating, body satisfaction and body image) outcomes for both groups over the short and long term. Acceptance was good for both programmes, but women attending the modified CBT programme were particularly positive about there being less focus on weight loss and more emphasis on self-acceptance. Indeed, it was the focus on emotional aspects of obesity that participants receiving the standard programme reported that they wanted more of.

Previously, these types of psychologically based programmes were the preserve of specialists, often delivered by psychologists or specially trained dietitians. One striking exception is the LEARN programme, first developed in the late 1970s [14], which was the first attempt to produce a treatment manual for individuals trying to lose weight or for practitioners working with overweight clients. However, no such programme was available in the UK. Jane appreciated that this issue of dissemination was hampering efforts to make gold standard treatments widely available and, in the light of evidence supporting self-help approaches [15], she undertook work to produce such a programme. Building on her previous work around modified CBT, Jane also felt it was essential that such a programme also address the emotional aspects of obesity and issues around body image. She therefore set about adapting the aforementioned modified CBT programme into a self-help format. Other work carried out by Jane and colleagues informed this work. For example, she had shown in an experimental study that emotional eaters increased their eating of unhealthy foods under stressful conditions [16]. She had also shown, using data from a large international survey, that low social support was independently associated with negative personal health behaviours, such as smoking, not eating breakfast and irregular sleep, in young men and women [17]. The resulting self-help programme was called Shape-Up; it was based on CBT approaches and incorporated modules on body image and size acceptance, managing overeating, social support, stress management, healthy eating and increasing physical activity [18].

Jane’s ongoing work in the field of obesity and weight management had also led her to establish the charity Weight Concern in 1997 to address the psychological as well as medical needs of overweight and obese individuals (www.weightconcern.org.uk). The charity provided a mechanism for her to undertake truly applied research and dissemination. One of the major achievements of the charity was to disseminate Shape-Up nationally through primary care and then local authority networks. Alongside the original programme described above was a briefer version [19•] and accompanying facilitator manual [20]. The facilitator manual used a peer support model to enable individuals to deliver Shape-Up in a group setting to provide additional support for people following Shape-Up. To date hundreds of facilitators have been trained, thousands have attended Shape-Up groups, and a number of local authorities deliver Shape Up as part of their routine weight management services. The approach has proved so popular that Jane and her group went on to adapt it for overweight adults with mild to moderate learning disabilities [21], and more recently for individuals who have completed treatment for cancer [22].

Jane also conducted important translational research in the treatment of childhood obesity. Despite the considerable research interest in treating adults with obesity, there was a dearth of evidence in children in the early noughties. Work from the USA had established ‘family-based behavioural treatment’ (FBBT) as the treatment of choice, but little work had been conducted outside this single research group [23]. Jane and colleagues adapted and piloted this programme for use in the UK with colleagues from Great Ormond Street Hospital. The programme comprised behavioural strategies including self-monitoring, goal setting, stimulus control, positive reinforcement and relapse prevention along with cognitive strategies for problem solving and to help manage teasing, alongside parenting strategies to support child behaviour change. For the pilot, the programme was delivered to 33 obese children, aged 8–12 years, each attending 12 sessions. Acceptance was good, with 27 children completing the programme and children significantly reduced their level of overweight and experienced improvements in psychological functioning over 3 months [24]. However, a subsequent randomised controlled trial comparing the programme (now extended to 6 months) to standard care found that, although children reduced their level of overweight over treatment, there was no difference between the two groups [25]. Despite this disappointment, it was at least encouraging that even the heaviest children in challenging circumstances could take steps to control their weight, and the study has contributed to the evidence base on childhood obesity treatments.

## Novel Approaches to Treating Obesity

Jane made well-established approaches more accessible to health professionals and individuals, but she did not stop there. She also developed and tested novel interventions and approaches. Jane applied her interest in the genetic aspects of obesity [26, 27] to her work on treatment approaches. Along with colleagues she looked at whether there was an additive effect on motivation to lose weight from providing personalised genetic feedback for weight control [28]. University students (*n* = 1016) were randomised to receive either weight loss advice alone or advice along with feedback from genetic testing. Participants who had received the genetic feedback had significantly higher levels of motivation to lose weight, as measured using the ‘Stages of Change’ questionnaire [29], than those receiving advice only [30•]. Levels of motivation overall were low, but tended to be highest in those who received higher-risk feedback and importantly, motivation was not reduced in those receiving low risk results. There was however no impact on behaviour change. This study did not specifically target overweight individuals or indeed those concerned about their weight, but using this type of approach in such a group was explored in another study using a qualitative methodology [31]. Participants in this study uniformly reported seeking an explanation for their struggles with weight control and those receiving a high-risk result found this reassuring. The feedback did not appear to make individuals complacent about managing their weight, instead they were motivated to overcome their genetic predisposition to gain weight. These findings are reassuring as they suggest that receiving genetic feedback could be helpful and not hinder weight management efforts.

In addition to exploring the application of genetic testing for weight control and developing behavioural interventions, Jane also recognised that other, simpler approaches could be useful at a public health level. For example, her pioneering work to apply habit formation theory to behaviour change interventions. ‘Habits’ are commonly referred to in weight management programmes but the habit model is rarely used formally. According to this model, habits are behaviours which are, through context consistent repetition, automatically triggered [32]. As these behaviours become increasingly automatic, they also become less conscious and so require less effort from the participant. Jane believed that such an approach could be particularly useful for weight management where long-term maintenance is notoriously difficult. Therefore, Jane and colleagues tested this in 104 overweight and obese adults who were randomised to either a habit-based weight management intervention (with either weekly or monthly monitoring) or a waiting list control group [33]. Target behaviours included choosing low-fat options and low-energy drinks and limiting sedentary time. Those who participated in the habit-based intervention lost significantly more weight (2.0 kg on average) over the 8-week intervention period compared to the control group, with the frequency of monitoring not significantly influencing weight loss. The intervention groups experienced further weight loss over the follow-up period of 32 weeks, but data were not available for the control group past 8 weeks. Participants reported that the target behaviours had become more automatic during the study and the level of automaticity was associated with greater weight loss.

Building on these encouraging results, this simple habit-based intervention was used as a basis for the ‘Ten Top Tips’ leaflet. This was produced by Jane in collaboration with the charities Cancer Research UK and Weight Concern (http://www.cancerresearchuk.org/about-cancer/causes-of-cancer/bodyweight-and-cancer/how-to-keep-a-healthy-weight). The leaflet includes 10 simple behaviours, outlined in Table 1, aimed at inducing a negative energy balance along with supporting information about the habit model and developing new habits. In order to test the hypothesis that such a low intensity approach could be effective for weight control at the population level, Jane and colleagues embarked on a large randomised controlled trial [34]. Adults with obesity were recruited through GP practices and randomised to either a ‘Ten Top Tips’ group or to receive ‘usual care’. This was ground breaking, insofar as it was the first such intervention to be delivered in primary care, and as a simple and brief approach, it was at odds with the more complex comprehensive programmes that dominate obesity care pathways.

**Table 1:** The Ten Top Tips

1	Keep to a meal routine—eat at roughly the same times each day
2	Go reduced fat—for foods like dairy products, spreads and salad dressings
3	Walk off the weight—10,000 steps per day
4	Pack a healthy snack—for example, fresh fruit instead of biscuits or crisps
5	Look at the labels—check the fat and sugar content on food labels
6	Caution with your portions—do not heap food on your plate and think twice before having second helpings
7	Up on your feet—break up sitting time
8	Think about your drinks—choose water or sugar-free squashes and limit fruit juice to one glass per day
9	Focus on your food—do not eat on the go
10	Do not forget your 5 a day—whether fresh, frozen or tinned

The Ten Tops Tips are simple weight loss tips; they are presented with supporting information about creating new habits based on habit formation theory. Accessible at http://www.cancerresearchuk.org/about-cancer/causes-of-cancer/bodyweight-and-cancer/how-to-keep-a-healthy-weight

Over the 3-month intervention period, those receiving the ‘Ten Top Tips’ (10TT) intervention lost significantly more weight than the ‘usual care’ group and experienced greater increases in automaticity, suggesting habits were being formed. Although the weight loss observed was small in absolute terms (mean 1.68 kg), at a population level, this has great potential [35•]. Importantly, this weight loss was also maintained at the 2 year follow-up, with almost a third of participants losing 5% or more of their initial weight. Although the ‘usual care’ group lost a similar amount of weight over 2 years, ‘usual care’ mostly consisted of referrals to more intensive community programmes such as Weight Watchers. This suggests that 10TT still holds promise as a low-cost, low intensity option within primary care. Jane’s work on habit-based interventions continues, with studies exploring the application of this approach in different groups and settings. For example, a randomised controlled trial is currently underway testing a habit-based intervention to improve diet and physical activity behaviours in patients with breast, colorectal and prostate cancer [36]. If successful, this intervention has the potential to be embedded within the cancer care pathway.

## Obesity Prevention and Health Promotion

Jane also applied habit formation theory to the field of obesity prevention in the context of encouraging healthy feeding habits in parents of young children. Previous research in this age group had typically focused simply on advice giving, and results had been inconsistent [37]. Based on the early work described above [33], Jane and colleagues developed a habit-based intervention for parents. This intervention aimed to encourage the uptake of healthy feeding behaviours in three target areas: serving fruit and vegetables/healthy snacks/non-sweetened drinks. It was evaluated within an exploratory cluster-randomised trial with parents of 2–6 year olds recruited from Children’s Centres in London [38•]. Parents randomised to receive the intervention had four home-based visits over an 8-week period. During these visits, parents were provided with information and tips on habit formation and the target behaviours, with a new habit being encouraged at each visit. There were significant group differences for the primary outcomes (automaticity scores for the 3 feeding behaviours) at follow-up and also higher reported intake of vegetables, healthy snacks and water in children. The intervention was well received with parents finding the intervention acceptable and easy to implement. A study in a subsample of participants using quantitative and qualitative data showed that the habit-formation model was well understood and considered useful [39]. Additionally, that study provided useful data regarding the process involved in forming new habits. For example, the types of goal set (e.g. the frequency, how specific the target) did not appear to substantially affect the likelihood of habit uptake and it was possible to follow more than one goal concurrently without any detrimental effects. Encouragingly, habit strength was shown to increase over the course of the intervention and then either stabilised or increased further by follow-up. This was the first study to offer an alternative approach for encouraging healthy and long-term changes in parents of young children and provide insights as to how habit formation could be optimised.

Jane also led independent evaluations of several public health campaigns run by other groups. For example, The Fighting Fat Fighting Fit campaign run by the BBC [40, 41] and Change4Life spearheaded by Public Health England [42]. Additionally, she was involved in the recent evaluation of the National Child Measurement Programme, also directed by Public Health England [43].

## Conclusions: Summing up

Despite Jane’s far reaching areas of research interest, the study of obesity was always a passion for her and she approached it with the same scientific vigour as all of her work. She challenged established views in the pursuit not only of science but to bring about improvements in treatments for adults and children. The significant contribution of Jane’s work both to discovery and applied science within public health has become apparent. She was very much concerned with how these findings might be applied in real-life settings and dissemination of successful programmes was facilitated through her charity Weight Concern. Jane’s legacy lies in both her research and these disseminated interventions, which continue to help individuals across the UK to manage their weight, improve their health and enhance their quality of life.
